# Exploring Peer Group and Cultural Influences on Substance Abuse Amongst School Learners in a Rural Community of Limpopo Province: A Qualitative Study

**DOI:** 10.3390/ijerph23070927

**Published:** 2026-07-20

**Authors:** Nancy Netshakhuma, Livhuwani Muthelo, Linneth Nkateko Mabila

**Affiliations:** 1 Department of Nursing Science, Faculty of Health Science, University of Limpopo, Sovenga, Private Bag X1106, Polokwane 0727, South Africa; nancynetshakhuma@gmail.com; 2 Department of Pharmacy, Faculty of Health Science, University of Limpopo, Sovenga, Private Bag X1106, Polokwane 0727, South Africa; nkateko.mabila@ul.ac.za

**Keywords:** adolescents, substance abuse, peer pressure, cultural influences, psychosocial support, rural communities, South Africa

## Abstract

**Highlights:**

**Public health relevance—How does this work relate to a public health issue?**

- Substance abuse and poor mental health among adolescents are increasing public health concerns, particularly in rural communities.
- Cultural practices and traditional beliefs play a significant role in shaping health behaviours in rural communities.

**Public health significance—Why is this work of significance to public health?**

- The study has the potential to strengthen mental health promotion, substance abuse prevention, and early intervention strategies among adolescents in rural schools.
- The study acknowledges the influence of cultural values and practices within rural communities.

**Public health implications—What are the key implications or messages for practitioners, policy makers and/or researchers in public health?**

- The findings may influence the development of policies and school health programmes aimed at improving adolescent mental health
- Addressing substance abuse and mental health challenges among adolescents therefore contributes not only to individual wellbeing but also to community development and public health improvement in rural South Africa.

**Abstract:**

Background: Adolescent substance abuse is a growing public health concern associated with poor mental health, unsafe sexual behaviour, violence, school dropout, and psychosocial dysfunction. In rural South Africa, peer influence, cultural practices, poverty, and inadequate psychosocial support may increase learners’ vulnerability to substance use. However, limited qualitative evidence exists on how these factors influence substance abuse among school learners in rural communities. Aim: This study explored peer-group and cultural influences on substance abuse among school learners in a rural community in Limpopo Province, South Africa. Methods: A qualitative, exploratory, descriptive, and contextual design was adopted. Purposive sampling was used to recruit 30 learners from selected secondary schools in the DIMAMO area of the Capricorn District, Limpopo Province. Data were collected through semi-structured one-on-one interviews until data saturation was reached. Interviews were audio-recorded, transcribed verbatim, and analysed using thematic analysis guided by Social Learning Theory. Trustworthiness was ensured through credibility, dependability, confirmability, and transferability. Ethical approval and informed consent were obtained prior to data collection. Results: Two major themes emerged: causes of substance abuse and factors maintaining substance use. Peer pressure and social influence contributed to the initiation of alcohol, cannabis, cigarette, and snuff use. Cultural practices and ancestral beliefs further normalised substance use behaviours. Psychological distress associated with poverty, family conflict, and inadequate parental support increased learners’ vulnerability. Continued substance use was reinforced by addiction, perceived emotional relief, social tolerance, and easy access to substances. Conclusions: Adolescent substance abuse in rural communities is influenced by interconnected social, cultural, psychological, and environmental factors. Integrated school- and community-based psychosocial interventions are needed to strengthen adolescent mental health and substance abuse prevention strategies.

## 1. Introduction 

Substance abuse among adolescents is a growing public health concern globally because of its association with poor mental health outcomes, violence, unsafe sexual behaviour, school dropout, and increased vulnerability to HIV infection and other sexually transmitted infections (STIs) [1]. Adolescence is a developmental stage characterised by emotional, psychological, and social changes that increase susceptibility to experimentation with alcohol, tobacco, cannabis, and other psychoactive substances [2]. Persistent substance use during adolescence negatively affects psychosocial development, educational attainment, and long-term health outcomes.

In South Africa, substance abuse among school-going learners remains a major educational and public health challenge. National reports indicate that experimentation with alcohol and drugs often begins during early adolescence, frequently before the age of 13 years [3]. Commonly abused substances among learners include alcohol, cigarettes, cannabis (“dagga”), and snuff [4]. Substance abuse among adolescents has been associated with poor academic performance, absenteeism, depression, aggression, unsafe sexual practices, and teenage pregnancy [5]. Recent South African studies indicate that substance use remains highly prevalent among school-going adolescents, with alcohol and cannabis being the most used substances. Evidence further suggests that male learners generally report higher rates of substance use than females; however, substance use among female adolescents has increased over recent years, narrowing the historical gender gap [6]. These gendered patterns highlight the need for interventions that address the unique social and behavioural factors influencing substance use among both male and female learners.

Rural communities are particularly vulnerable because of poverty, unemployment, limited recreational opportunities, and inadequate psychosocial support services [5]. Learners residing in rural communities may face additional vulnerabilities that increase their risk of substance abuse. Limited access to mental health services, educational resources, recreational facilities, and psychosocial support programmes may contribute to increased exposure to substance use and other risk-taking behaviours [7]. Rural communities in Limpopo Province continue to experience socio-economic challenges that may heighten adolescents’ susceptibility to peer pressure and substance abuse.

The Social Learning Theory provides a relevant framework for understanding adolescent substance abuse, as it explains how behaviours are learned through observation and social interaction [8,9]. Peer influence remains one of the strongest predictors of adolescent substance use, with learners often initiating substance use to gain social acceptance and social belonging [10]. Cultural beliefs and traditional practices may also contribute to substance use behaviours among adolescents in some rural communities, particularly where substances such as snuff are associated with ancestral rituals and traditional healing practices [11,12]. The relationship between substance abuse, poverty, trauma, and mental health among adolescents has received increasing attention in recent years. Poverty-related stressors such as food insecurity, family instability, parental unemployment, and exposure to community violence may contribute to emotional distress and increased vulnerability to substance use [13]. Furthermore, individuals who have experienced childhood trauma often exhibit low self-esteem and are at an increased risk of developing depression and anxiety. Some may deny or suppress their traumatic experiences, while others may construct a false self-image as a coping mechanism. Additionally, alcohol and substance misuse are frequently adopted as maladaptive strategies to avoid confronting or managing the psychological consequences of their traumatic experiences [14]. These findings suggest that adolescent substance abuse should be understood not only as a behavioural problem but also as a mental health and social development concern.

In addition, psychological distress associated with family conflict, parental loss, trauma, and emotional stress may increase adolescents’ vulnerability to substance abuse as a coping mechanism [15]. South Africa’s National Drug Master Plan (NDMP) 2019–2024 advocates a comprehensive and evidence-based approach to preventing substance abuse through collaboration among schools, families, communities, and government sectors [16]. Similarly, the Health Sector Drug Master Plan (2019–2024) emphasises strengthening prevention programmes, improving access to treatment services, and providing psychosocial support for vulnerable populations affected by substance use [17]. Similarly, the Integrated School Health Policy advocates promoting mental health and psychosocial well-being among school-going learners [18].

Although previous studies have documented the prevalence, determinants, and consequences of adolescent substance abuse in South Africa, much of the existing evidence is derived from quantitative studies that focus primarily on urban settings or broad adolescent populations [6,7]. While peer influence and cultural factors have been identified as important determinants of substance use among adolescents [10,11], limited qualitative research has explored how these factors interact to shape substance use behaviours among school learners in rural communities. Furthermore, there is a paucity of context-specific evidence from rural Limpopo Province regarding learners’ lived experiences of peer pressure, social acceptance, cultural norms, and traditional practices related to substance use. This knowledge gap limits the development of culturally appropriate and contextually relevant prevention interventions. Therefore, this study seeks to explore peer-group and cultural influences on substance abuse among school learners in a rural community in Limpopo Province, South Africa. Understanding learners’ lived experiences may contribute to the development of culturally sensitive interventions aimed at reducing substance abuse and promoting adolescent mental health and psychosocial well-being.

## 2. Materials and Methods

### 2.1. Study Design

This study employed a qualitative, exploratory, descriptive, and contextual research design to examine peer-group and cultural influences on substance abuse among school learners in a rural community in Limpopo Province, South Africa. Qualitative research enables researchers to explore and understand participants’ lived experiences, perceptions, and meanings within their natural settings [19,20]. The exploratory and descriptive nature of the study enabled an in-depth understanding of learners’ experiences with substance abuse, while the contextual approach ensured that the findings were interpreted in light of the realities of the rural school environment [21].

The study was further informed by Bandura’s Social Learning Theory, which explains that behaviour is learned through observation, imitation, and interaction within social environments [8,9]. The theory was considered relevant because it provided a framework for understanding how peer influence, social interaction, and cultural practices contribute to adolescents’ substance use behaviours.

### 2.2. Study Setting

The study was conducted in selected secondary schools within the DIMAMO Health and Demographic Surveillance Site (HDSS) situated in the Capricorn District, Limpopo Province, South Africa. The DIMAMO area consists predominantly of rural and semi-rural communities comprising approximately 51 villages and 21 secondary schools. The population is mainly Sepedi-speaking and experiences socio-economic challenges such as poverty, unemployment, substance abuse, and limited access to psychosocial support services. The setting was considered appropriate because it provided the researchers with an opportunity to explore learners’ experiences regarding substance abuse within their real social and cultural environments.

### 2.3. Population and Sampling

The study population comprised school-going learners from selected secondary schools in the DIMAMO area. Learners in Grades 8 to 12 who had experience with or knowledge of substance abuse among learners were eligible to participate in the study. Specifically, participants included learners who reported personal experience with substance use, learners who had close peer relationships with substance users, and learners who had directly observed substance use behaviours among learners within the school environment. Including participants with diverse experiences enabled the researchers to obtain a comprehensive understanding of peer-group and cultural influences on substance abuse.

A non-probability purposive sampling technique was used to recruit information-rich participants who could provide detailed insights into peer group and cultural influences on substance abuse [21]. Seven secondary schools were selected from the 21 schools within the DIMAMO area. Following approval from the Limpopo Department of Education and school principals, the researcher conducted information sessions with eligible learners to explain the study’s purpose, participation requirements, ethical considerations, and participants’ rights. Teachers and school management teams assisted only in coordinating recruitment activities and identifying suitable venues and times for interviews; they were not involved in selecting participants. Learners who expressed interest in participating voluntarily contacted the researcher and were screened against the inclusion criteria. Participation was entirely voluntary, and learners were informed that their decision to participate or decline would not affect their academic standing or relationship with the school. Written informed consent was obtained from participants aged 18 years and older, while parental consent and learner assent were obtained for participants younger than 18 years. Data saturation was reached at participant number 30, after which no new themes or information emerged.

### 2.4. Research Team and Reflexivity

Data were collected by the primary researcher, a female professional nurse with experience in mental health and adolescent health research. Although the researcher had prior knowledge of substance abuse challenges within rural communities, reflexive practices were employed to minimise bias during data collection and analysis. Reflexive journaling, memo writing, and continuous discussions with supervisors were used throughout the research process to bracket personal assumptions and ensure that findings remained grounded in participants’ lived experiences [21].

The researcher had no prior relationship with the participating learners, which reduced the possibility of role-related influence during interviews. Field notes were also used to document contextual observations and nonverbal cues during interviews.

### 2.5. Data Collection

Data were collected through semi-structured one-on-one interviews conducted between learners and the primary researcher in private rooms within the school premises to ensure privacy, confidentiality, and free expression. Semi-structured interviews allowed flexibility and enabled participants to provide detailed accounts of their experiences regarding substance abuse among learners [21,22].

One central opening question guided the interviews: “Can you describe your experience regarding substance abuse among learners in your school?” Additional probing questions included: “How do friends influence learners to use substances?” “What cultural beliefs or practices do you think influence substance use among learners?” “How does substance use affect learners’ relationships and school performance?”, and “What challenges do learners who use substances experience?” These probing questions facilitated a deeper exploration of participants’ experiences and perceptions. The interview guide has been provided as Supplementary Material File S1. Interviews were conducted in English and in local languages preferred by participants and lasted approximately 25–35 min.

With participants’ permission, all interviews were audio-recorded to ensure accurate capturing of information. Field notes were also taken to document non-verbal communication, emotional responses, and contextual observations. Interviews conducted in local languages were transcribed verbatim and translated into English by a bilingual language expert. To ensure linguistic accuracy and preserve participants’ intended meanings, translated transcripts were independently reviewed by a second bilingual reviewer and compared with the original audio recordings. Any discrepancies were discussed and resolved by consensus, thereby ensuring conceptual equivalence and preserving cultural nuances throughout the analysis [23]. A pilot interview was conducted with four learners who were excluded from the final study to refine the interview guide and improve interviewing skills. Data collection continued until saturation was reached at participant number 28, as confirmed by the last two participants, for a total of 30 participants.

### 2.6. Data Analysis

Data were analysed using thematic analysis following the procedures described by [22]. Audio-recorded interviews were transcribed verbatim and translated into English where necessary. Prior to analysis, translated transcripts were reviewed against the original recordings by bilingual reviewers to verify translation accuracy and ensure that participants’ meanings, expressions, and contextual nuances were retained. The researchers familiarised themselves with the data by repeatedly reading transcripts to gain immersion in and understanding of participants’ experiences.

Initial codes were generated systematically by identifying meaningful statements related to peer group and cultural influences on substance abuse. Similar codes were grouped into categories, which informed the development of themes and sub-themes through constant comparison across transcripts. Themes were refined collaboratively by the researchers to ensure they accurately reflected participants’ experiences. Verbatim quotations were used to support the identified themes and preserve the authenticity of participants’ voices.

To enhance credibility, an independent coder experienced in qualitative research analysed a sample of transcripts, and consensus discussions were held on coding and theme development [21].

### 2.7. Measures to Ensure Trustworthiness

Trustworthiness was ensured using Lincoln and Guba’s criteria of credibility, dependability, confirmability, and transferability [24]. Credibility was enhanced through prolonged engagement with participants, member checking, audio recording of interviews, triangulation of interview data with field notes, and verification of translated transcripts through independent review. Dependability was ensured by maintaining a detailed audit trail documenting methodological decisions, coding processes, and theme development.

Confirmability was strengthened through reflexive journaling, independent coding, and consensus discussions among the researchers to minimise bias. Transferability was facilitated through detailed descriptions of the study setting, participants, sampling process, and research methodology, enabling readers to assess the applicability to similar contexts.

### 2.8. Ethical Considerations

Ethical approval was obtained from the University of Limpopo Turfloop Research Ethics Committee prior to data collection. Permission to conduct the study was also obtained from the Limpopo Department of Education and participating schools. The study adhered to ethical principles of autonomy, beneficence, non-maleficence, and justice [19].

Participation was voluntary, and participants were informed of the study’s purpose, procedures, potential risks, and their right to withdraw at any stage without penalty. Confidentiality and anonymity were maintained by using participant codes instead of names. All data, including transcripts and audio recordings, were stored securely on password-protected devices accessible only to the research team.

## 3. Results

### 3.1. Demographic Profile of Participants

The study included 30 school-going learners from selected secondary schools in the DIMAMO area of the Capricorn District, Limpopo Province. Participants were between 14 and 19 years of age and were recruited from Grades 8 to 12. Both male and female learners participated in the study. Participants provided rich descriptions regarding peer group and cultural influences on substance abuse among learners within their schools and communities.

### 3.2. Themes and Sub-Themes

Data analysis resulted in the emergence of two major themes and related sub-themes regarding peer group and cultural influences on substance abuse among school learners (Table 1). The findings revealed that substance abuse among learners is influenced by a complex interaction of peer-group dynamics, cultural and family environments, psychological distress, and broader community factors. Participants described factors contributing to the initiation of substance use, as well as factors that sustain continued substance use among adolescents.

The findings are presented according to the identified themes and sub-themes. Verbatim quotations are included to preserve the authenticity of participants’ experiences and perceptions regarding substance abuse among learners.

#### 3.2.1. Theme 1: Causes of Substance Abuse Among Learners

Participants described several factors contributing to substance abuse among learners within rural communities. These included peer pressure, cultural practices, emotional distress, and family-related challenges. Learners indicated that substance use often begins during adolescence because of the desire for social acceptance, curiosity, and exposure within community environments.

##### Sub-Theme 1.1: Peer Pressure and Social Influence

Participants identified peer pressure as one of the strongest contributors to substance abuse among learners. Learners described how friendship groups and social networks influence experimentation with alcohol, cigarettes, cannabis, and other substances. Participants explained that many learners engage in substance use to gain acceptance, appear “fashionable,” or avoid social exclusion.

Participants described peer influence as a major driver of substance abuse among learners. Participant 4 stated, “Most learners start smoking because of friends. If you are always with friends who smoke dagga or cigarettes, they convince you to try it, and later you become used to it.” Similarly, Participant 11 explained, “Sometimes you smoke because you want to fit in the group. If you refuse, they laugh at you and say you are weak or childish.” Participant 18 further added, “Others are forced by peer pressure at school. You find learners smoking behind classrooms, and if you are always around them, you end up doing the same thing.”

Participants’ accounts suggested that peer influence extended beyond direct pressure to include the modelling of behaviours, social reinforcement, and a desire to maintain a sense of belonging. Learners described substance use as a behaviour that often became normalised within friendship groups, making experimentation more likely among adolescents seeking acceptance and identity within their peer networks.

Participants further indicated that peer influence was common during social gatherings, sporting activities, and after-school interactions where learners are exposed to substance use behaviours.

##### Sub-Theme 1.2: Cultural and Traditional Influences

Participants described how cultural beliefs and traditional practices contributed to substance use behaviours among some learners. Learners reported that substances such as snuff and alcohol are sometimes used during ancestral rituals, traditional ceremonies, and healing practices within their communities.

Participants highlighted that cultural practices and exposure to substance use within family environments contributed to the normalisation of substance use behaviours among adolescents. Participant 7 stated, “At home, they use snuff when communicating with ancestors, so some children become curious and start using it even when elders are not around.” Similarly, Participant 15 explained, “In our community, alcohol is used in traditional ceremonies, and young people see adults drinking all the time, so they think it is normal.” Participant 21 further added, “Some learners believe smoking makes them strong because they see older people doing it during traditional gatherings.”

The findings suggest that cultural exposure did not necessarily encourage substance use directly; rather, repeated observation of substance use within culturally accepted settings appeared to influence learners’ perceptions of what constitutes acceptable behaviour. Participants described how cultural and family environments may contribute to the normalisation of certain substances among adolescents. Participants indicated that exposure to substance use within cultural and family environments contributed to early experimentation among adolescents.

##### Sub-Theme 1.3: Psychological and Family-Related Factors

Participants reported that emotional distress, family conflict, poverty, and lack of parental support contributed to substance abuse among learners. Some learners described using substances as a coping mechanism for stress, emotional pain, and difficult home environments.

Participants associated substance abuse with emotional and family-related challenges experienced by learners. Participant 9 stated, “Some learners smoke or drink because of stress at home. Maybe parents are always fighting, or there is no peace at home.” Participant 13 explained, “Others use drugs because they feel lonely after losing parents or because nobody cares about them.” Participant 24 added, “Poverty also causes substance abuse because some learners think drugs help them forget their problems.”

Participants frequently linked substance use to experiences of emotional hardship and social adversity. Family instability, inadequate parental supervision, bereavement, and financial difficulties were described as circumstances that increased learners’ vulnerability to experimenting with substances as a means of coping with distressing life situations. Participants further indicated that a lack of parental supervision and poor communication within families increased learners’ vulnerability to substance use behaviours.

#### 3.2.2. Theme 2: Factors Maintaining Substance Abuse Among Learners

Participants described several factors that contributed to the continuation of substance use after initiation. These factors included addiction and dependency, perceived positive effects of substances, and easy access to substances within schools and communities.

##### Sub-Theme 2.1: Addiction and Dependency

Participants reported that continued substance use was often influenced by addiction and dependency. Learners explained that after repeated use, some individuals found it difficult to stop using substances despite being aware of the harmful consequences.

Participant 5 stated, “Some learners want to stop smoking, but they cannot because their bodies are already used to it.” Participant 12 explained, “When they do not smoke or drink, they become angry or uncomfortable, so they go back to using it again.” Similarly, Participant 27 noted, “Others know that drugs are destroying them, but they keep taking them because they feel they cannot live without them.”

Participants described dependency as a cycle in which initial experimentation gradually progressed into habitual use. According to learners, repeated substance use created strong cravings and emotional reliance on substances, making cessation difficult even when learners recognised the negative effects on their health, education, and relationships.

##### Sub-Theme 2.2: Perceived Positive Effects of Substances

Participants reported that learners continue using substances because they believe substances help them cope with stress, improve confidence, and provide temporary relief from emotional problems.

Participants described perceived emotional and social benefits as reasons for continued substance use among learners. Participant 2 stated, “Some learners believe alcohol helps them forget stress and family problems.” Similarly, Participant 14 explained, “Others say smoking makes them feel relaxed and confident when they are around friends.” Participant 20 further added, “Learners think drugs help them escape reality, especially when life is difficult at home.”

Participants indicated that these perceived benefits often reinforced continued use. Temporary feelings of relaxation, confidence, and emotional relief appeared to strengthen the likelihood of repeated substance use despite awareness of associated risks. Participants indicated that these perceived positive effects contributed to repeated substance use despite awareness of harmful consequences.

##### Sub-Theme 2.3: Easy Access and Social Tolerance of Substances

Participants described easy access to substances within communities and schools as an important factor in maintaining substance abuse among learners. Learners reported that substances were easily available through peers, local taverns, and community members.

Participants highlighted that easy availability and community exposure contributed to continued substance abuse among adolescents. Participant 6 stated, “Learners easily get alcohol from taverns because some adults buy for them.” Participant 17 explained, “Some learners even bring cigarettes and drugs to school and sell them to others.” Participant 25 added, “In the community, substance abuse is common, so young people see it as something normal.”

The findings further suggest that the availability of substances within communities, coupled with perceived social tolerance of substance use, created an environment in which continued use was facilitated and reinforced. Participants described how widespread community exposure to substance use reduced perceived risks and increased opportunities for access among learners. Participants further highlighted that weak parental supervision and limited law enforcement contributed to the continued availability of substances among adolescents.

## 4. Discussion

This study explored peer-group and cultural influences on substance abuse among school learners in a rural community of Limpopo Province, South Africa. The findings demonstrate that adolescent substance abuse is not the result of a single risk factor but rather emerges from the interaction of social, cultural, psychological, and structural influences within learners’ everyday environments. The findings suggest that peer-group dynamics, cultural exposure to substances, family-related distress, and socio-economic challenges collectively create conditions that increase both the initiation and maintenance of substance use among adolescents.

Peer pressure and social influence emerged as important contributors to substance abuse among learners. Participants described how adolescents often initiate substance use to gain acceptance, maintain friendships, and avoid social exclusion. These findings support Bandura’s Social Learning Theory, which proposes that behaviours are learned through observation, imitation, and reinforcement within social contexts [8]. Similar findings have been reported in studies showing that adolescents whose peers use substances are more likely to engage in substance use themselves because such behaviours become normalised within friendship networks [6,10]. However, the current study extends existing knowledge by demonstrating that peer influence operates not only through direct pressure but also through subtle processes of social belonging, identity formation, and behavioural modelling. Learners appeared to associate substance use with social acceptance and status within peer groups, suggesting that prevention programmes should address social norms and group influences rather than focusing solely on individual behaviour change.

The findings further revealed that cultural beliefs and traditional practices may indirectly influence adolescent substance use. Participants described exposure to substances such as alcohol and snuff during traditional ceremonies, ancestral rituals, and family gatherings. Similar observations have been reported in African contexts where alcohol and other substances are incorporated into cultural and social practices [11]. Importantly, the findings do not suggest that cultural practices cause substance abuse. Rather, they indicate that repeated exposure to substance use within culturally accepted settings may contribute to the perception that certain substances are socially acceptable or harmless. This distinction is important because it highlights the need for culturally sensitive interventions that respect traditional practices while simultaneously promoting awareness regarding the risks associated with adolescent substance use.

Psychological distress and family-related challenges also emerged as significant contributors to substance abuse among learners. Participants linked substance use to family conflict, bereavement, emotional distress, inadequate parental support, and poverty. These findings are consistent with previous studies indicating that adolescents experiencing psychosocial stressors are more likely to engage in substance use as a coping mechanism [13,25]. The findings suggest that substance use among adolescents may function as a maladaptive response to emotional pain and unresolved psychosocial challenges. Consequently, substance abuse should not be viewed solely as a behavioural problem but also as a mental health and social welfare concern requiring integrated psychosocial support.

An important contribution of this study is the identification of how peer influence, cultural exposure, and family distress interact within the broader context of rural disadvantage. Participants frequently described poverty, unemployment, inadequate supervision, and limited opportunities for recreation as underlying conditions that increased learners’ vulnerability to substance use. These findings suggest that structural vulnerabilities create environments in which peer pressure and substance availability exert stronger influence. In this regard, substance abuse may be understood as a product of both individual experiences and broader socio-economic conditions that shape adolescents’ daily lives.

The study also identified addiction and dependency as important factors maintaining substance use among learners. Participants described how learners continued using substances despite recognising their harmful effects. This finding highlights the progression from experimentation to habitual use, illustrating that the factors initiating substance use may differ from those sustaining it. While peer influence and curiosity may contribute to initial experimentation, dependency appears to reinforce continued use over time. This observation supports existing evidence indicating that repeated substance use can create psychological and physiological dependence that complicates cessation efforts among adolescents [26].

Participants further reported that learners continued using substances because they perceived them to provide emotional relief, relaxation, confidence, and temporary escape from stressful circumstances. Similar findings have been reported in studies linking substance use to self-medication and coping behaviours among adolescents experiencing emotional distress [13]. The findings therefore suggest that interventions focusing exclusively on substance abuse prevention may be insufficient unless they simultaneously address underlying emotional and mental health needs. School-based mental health promotion and counselling services may play an important role in reducing adolescents’ reliance on substances as coping mechanisms.

Easy access to substances and community tolerance of substance use were also identified as factors maintaining substance abuse. Participants described the availability of alcohol, cigarettes, and drugs through peers, community members, and local establishments. This finding reflects the influence of the broader social environment on adolescent behaviour. When substances are easily accessible, and substance use is perceived as common within communities, adolescents may perceive such behaviours as normal and acceptable. The findings, therefore, highlight the importance of strengthening community-level prevention initiatives, parental monitoring, enforcement of regulations that restrict underage access to substances, and collaboration among schools, families, healthcare services, and community stakeholders.

Compared with previous studies that have primarily quantified prevalence rates and risk factors, this study provides a qualitative understanding of how multiple influences converge within the lived experiences of learners in a rural South African context. The findings demonstrate that peer pressure, cultural exposure, emotional distress, poverty, and community environments do not operate independently; rather, they interact and reinforce one another to shape substance use behaviours. This insight contributes to the existing body of knowledge by highlighting the complex and context-specific pathways through which substance abuse develops and is sustained among adolescents in rural communities.

## 5. Limitations of the Study

The study was conducted in selected secondary schools within a single rural community in Limpopo Province; therefore, the findings may not be generalisable to all school settings in South Africa. In addition, the study relied on self-reported experiences from learners, which may have been influenced by recall bias or reluctance to disclose sensitive information related to substance abuse.

## 6. Conclusions and Recommendations

Substance abuse among school learners in this rural Limpopo community is influenced by interconnected peer, cultural, psychological, family, and socio-economic factors. Peer pressure, cultural exposure to substances, emotional distress, family challenges, addiction, and easy access to substances contribute to both the initiation and continuation of substance use.

The findings highlight the need for integrated school- and community-based interventions that strengthen mental health support, life-skills development, substance abuse prevention, and family involvement. Collaboration among schools, families, healthcare professionals, and community stakeholders is essential to address the underlying factors contributing to adolescent substance abuse and promote healthier outcomes among learners.

## Figures and Tables

**Table 1:** Summary of Emergent Themes and Sub-Themes.

Themes	Sub-Themes
1. Causes of substance abuse among learners	1.1 Peer pressure and social influence
	1.2 Cultural and traditional influences
	1.3 Psychological and family-related factors
2. Factors maintaining substance abuse among learners	2.1 Addiction and dependency
	2.2 Perceived positive effects of substances
	2.3 Easy access and social tolerance of substances

## Data Availability

The data supporting the findings of this study are available from the corresponding author upon reasonable request.

## Supplementary Materials

The following supporting information can be downloaded at https://www.mdpi.com/article/10.3390/ijerph23070927/s1, File S1. Consolidated Criteria for Reporting Qualitative Research (COREQ) Checklist.
