# Supplementary material for: Exploring Peer Group and Cultural Influences on Substance Abuse Amongst School Learners in a Rural Community of Limpopo Province: A Qualitative Study
Source: Int J Environ Res Public Health. 2026 Jul 20;23(7):927. doi: 10.3390/ijerph23070927 (PMC13410280; doi:10.3390/ijerph23070927)
Supplement: Supplementary file 1 [file ijerph-23-00927-s001.zip › ijerph-4343727-supplementary.pdf]

File S1. Consolidated Criteria for Reporting Qualitative Research (COREQ) Checklist

Manuscript Title: *Exploring Peer Group and Cultural Influences on Substance Abuse amongst School Learners in a Rural Community of Limpopo Province: A Qualitative Study*

No.	Domain / Item	Guide Questions / Description	Reported in Manuscript
**Domain 1: Research Team and Reflexivity**			
1	Interviewer/facilitator	Which author conducted the interviews?	The primary researcher conducted all interviews.
2	Credentials	What were the researcher's credentials?	Professional nurse with experience in mental health and adolescent health-related research.
3	Occupation	What was the researcher's occupation at the time of the study?	Professional nurse/researcher.
4	Gender	Was the researcher's gender reported?	Female researcher
5	Experience and training	What experience or training did the researcher have?	Experience in mental health and adolescent health-related research was reported.

6	Relationship established	Was a relationship established prior to study commencement?	No prior relationship with participants was reported.
7	Participant knowledge of the interviewer	What did participants know about the researcher?	Participants were informed about the study purpose, ethical considerations, and voluntary participation.
8	Interviewer characteristics	What characteristics were reported about the interviewer/facilitator?	Reflexive journaling, memo writing, and supervision discussions were used to minimise bias and bracket assumptions.
**Domain 2: Study Design**			
9	Methodological orientation and theory	What methodological orientation underpinned the study?	Qualitative, exploratory, descriptive, and contextual design informed by Bandura's Social Learning Theory.
10	Sampling	How were participants selected?	Purposive sampling was used.
11	Method of approach	How were participants approached?	Through school management teams, after permission from the authorities.
12	Sample size	How many participants were included?	30 learners participated.
13	Non-participation	How many people refused to participate or dropped out?	None

14	Setting of data collection	Where was the data collected?	Private rooms within school premises in selected secondary schools in the DIMAMO area, Limpopo Province.
15	Presence of non-participants	Was anyone else present besides participants and researchers?	None
16	Description of sample	What are the important characteristics of the sample?	Learners aged 14–19 years from Grades 8–12; both male and female learners participated.
17	Interview guide	Were questions/prompts provided and pilot tested?	A semi-structured interview guide was used; a pilot interview was conducted with four learners, who were excluded from the final study.
18	Repeat interviews	Were repeat interviews carried out?	28 interviews, confirmed with 2 for a total of 30
19	Audio/visual recording	Did the research use audio or visual recording?	Interviews were audio-recorded with permission.
20	Field notes	Were field notes made during/after interviews?	Yes, field notes documented non-verbal communication and contextual observations.
21	Duration	What was the duration of the interviews?	Approximately 25–35 minutes.

22	Data saturation	Was data saturation discussed?	Yes, saturation was reached at participant 28 and confirmed with the final two participants.
23	Transcripts returned	Were transcripts returned to participants for comment/correction?	No transcript returned
**Domain 3: Analysis and Findings**			
24	Number of data coders	How many data coders coded the data?	Researchers analysed data collaboratively; an independent coder analysed a sample of transcripts.
25	Description of coding tree	Did the authors provide a description of the coding tree?	A table of themes and sub-themes was presented.
26	Derivation of themes	Were themes identified in advance or derived from the data?	Themes were derived inductively from thematic analysis.
27	Software	What software, if applicable, was used to manage the data?	No software used
28	Participant checking	Did participants provide feedback on findings?	Member checking was reported to enhance credibility.
29	Quotations presented	Were participant quotations presented to illustrate themes?	Yes, verbatim quotations were used to support themes and sub-themes.

30	Data and findings consistent	Was there consistency between the data presented and the findings?	Yes, findings were supported by participant quotations and thematic descriptions.
31	Clarity of major themes	Were major themes clearly presented?	Yes, two major themes and related sub-themes were clearly presented.
32	Clarity of minor themes	Is there a description of diverse cases or minor themes?	Minor/diverse cases were briefly presented
